# Experiences and challenges of acute coronary syndrome patients in care provision: a qualitative systematic review

**DOI:** 10.1186/s13643-024-02578-1

**Published:** 2024-07-17

**Authors:** Daniel Ameen, Kate Kynoch, Hanan Khalil

**Affiliations:** 1 Faculty of Medicine, Nursing and Health Sciences, Monash School of Medicine, Monash University, Melbourne, Australia; 2 Mater Health, Queensland University of Technology (School of Nursing), Queensland Centre for Evidence-Based Nursing and Midwifery: A JBI Centre of Excellence, Brisbane, QLD Australia; 3 Department of Public Health, School of Psychology and Public Health, La Trobe University, Melbourne, Australia

**Keywords:** Cardiovascular disease, Patients experience, Qualitative systematic review, Care provision

## Abstract

**Background:**

Coronary artery disease including acute coronary syndrome (ACS) constitutes the most common cause of death in people with cardiovascular disease. Prompt diagnosis and early initiation of treatment significantly impact on patient outcomes. Positive patient experience with their initial care is linked to positive clinical outcomes.

**Objective:**

This qualitative review aimed to investigate patients’ experience of care provision and the challenges faced by them during their different stages of care following an ACS.

**Methods:**

Searches of four databases - MEDLINE, Embase, CINAHL and PsychINFO - were conducted from inception until July 13, 2022, and were limited to English-language publications. Assessment of methodological quality of studies was performed using the Jonna Briggs Institute (JBI) qualitative assessment and review instrument. Data were extracted using the standardised data extraction tool from JBI. Data synthesis following the JBI approach of meta-aggregation was performed. The level of confidence for each synthesised finding was established based on ConQual.

**Results:**

Overall, from 578 records, 10 studies were included with 39 findings extracted from the included studies. The main synthesised findings were the need to provide tailored information and appropriate management at different stages of care, and that timely management and trust in health care workers are associated with greater patient satisfaction and more positive experiences.

**Conclusion:**

Patients with an ACS experience many challenges during different stages of their care. Clinicians should be aware of the challenges they face and provide tailored information to patients that is appropriate for their different stages of management in order to best optimise patient experience and improve patient outcomes.

**Supplementary Information:**

The online version contains supplementary material available at 10.1186/s13643-024-02578-1.

## Introduction

Ischaemic heart disease is associated with the highest comorbidity and mortality globally and account for more than 35% of world-wide death [1]. Moreover, according to a recent report published by the World Health Organization, 17.9 million people died in 2019 of ischaemic heart disease [2]. Acute coronary syndrome (ACS), a subset of ischaemic heart disease, describes any condition resulting in an acute disruption of blood flow to the myocardium, potentially resulting in tissue death. ACS constitutes the most common cause of death in people with cardiovascular disease [1]. Successful management of ACS depends on many factors including prompt diagnosis and early initiation of treatment which may vary significantly due to staffing, resources, adequacy of pain management and hospital environment [3].

Patient experience and patient satisfaction, while distinct concepts, are closely linked within healthcare and are well recognised and reported indicators for assessing the quality of healthcare [4]. Patient satisfaction is an outcome measure that impacts directly on a patient’s experience with a healthcare organisation [4]. Many healthcare providers regard patient experience as a core component of healthcare quality.

Following an ACS, patients’ needs evolve during their care depending on their individual experience, plan of care and the complexity of their condition [4, 5]. Studies have explored patient experience following a cardiac event to gain a deeper understanding of the needs and challenges faced by these patients. Several published studies have found that higher patient satisfaction with their management and care was associated with positive clinical outcomes, increased patient safety, and better use of hospital resources [6–8]. Further research also suggests that other factors such as patients’ expectations, characteristics, understanding of the health care system and the broader society are also contributing factors to their satisfaction and overall experience of their care [9].

To date, several studies have been published exploring patients’ satisfaction and overall experience with their care and management following an ACS [8–12]. These studies also report on the challenges and barriers patients face during their healthcare journey [10–14]. Healthcare staff can respond to patients' concerns which may result in increased patient satisfaction and an overall positive experience with their care leading to improved health outcomes [9]. This systematic review investigated patients’ experience of care provision and the challenges faced by them during their different stages of care when experiencing an ACS.

## Materials and methods

### Method

The review was conducted in accordance with guidance from JBI methodology for systematic reviews of qualitative evidence [15]. This review was conducted according to a priori protocol registered and published in PROSPERO (CRD42022346910).

### Inclusion criteria

#### Types of participants

- Patients admitted to hospital, regardless of age, due to an ACS (includes unstable angina, non-ST elevation myocardial infarction (NSTEMI), ST elevation myocardial infarction (STEMI))
- Patients reported on their experience with the care they were provided in an inpatient setting

#### Phenomenon of interest

The phenomena of interest for this review were the experiences with the provision of care provided and any challenged faced by them after an acute coronary syndrome.

#### Context

The context for this review was settings where patients received their initial healthcare following an ACS including their management in hospitals as an inpatient.

#### Types of studies

The review considered qualitative studies including, but not limited to, designs such as phenomenology, grounded theory and ethnography.

#### Patient and public involvement

No patient was involved in the design, planning and conception of this study.

### Search strategy

The search strategy aimed to find both published and unpublished grey literature. A three-step search strategy was undertaken in this review. An initial limited search of MEDLINE was conducted and followed by analysis of the text words contained in the title and abstract and of the index terms used to describe an article. A second search using all identified keywords and index terms was undertaken across all included databases. We matched the keywords, MeSH terms and thesaurus results related to the concepts of myocardial infarction or ST elevation myocardial infarction or non-ST elevation myocardial infarction or acute coronary syndrome, patients’ experience or satisfaction and health care or medical care or acute care or hospital care or primary care or healthcare services in each database. The full search strategies are provided in Supplementary material. Finally, the reference lists of included articles were hand searched for additional studies. The timeframe for included studies was from the inception of all databases until 13th July 2022. Due to lack of translation resources, only studies published in English language were included.

#### Information sources

The following databases were searched: MEDLINE, Embase, CINAHL and PsycINFO. The search for grey literature was undertaken in Google Scholar.

### Study selection

Following the search, all identified citations were collated and uploaded into EndNote (Version X9 (Clarivate Analytics, PA, USA), The duplicated citations were removed. Two independent reviewers (DA and HK) screened the title and abstract of each paper against the review’s inclusion criteria. Studies identified as potentially eligible or those without an abstract were retrieved for full-text review, and their details were imported into the JBI System for the Unified Management, Assessment and Review of Information (JBI SUMARI) [16]. Full texts that did not meet the review inclusion criteria were excluded. Any disagreements between the two reviewers were resolved through discussion.

### Assessment of methodological quality

Qualitative papers selected for inclusion were assessed by two independent reviewers (DA and HK) according to the 10-item checklist for qualitative research from Lockwood et al. (see Appendix 1 for checklist) [15]. The assessment evaluated methodology and philosophical perspective of the research, the research objectives, the methods used to collect data, analysis of data and the interpretation of results, if a statement of the influence of the researcher on the research was included and representation of the participants voices. All items were scored as either ‘yes’, ‘no’ or ‘unclear’ All papers were included in the review regardless of methodological quality. Any disagreements that arose between the reviewers were resolved through discussion.

### Data extraction

Two reviewers (DA and HK) independently extracted data from papers by using the standardised data extraction tool from JBI SUMARI. The extracted data included specific details about the study population, context, culture, geographical location, study methods and the phenomena of interest relevant to the review question (i.e. experiences of MI patients during provision of care and challenges faced by them). Findings and their illustrations were extracted and assigned a level of credibility [15].

### Data synthesis

Qualitative research findings were aggregated in JBI SUMARI using a meta-aggregation approach. The process to synthesise followed a three-step approach. First, individual extracted study findings were rated as either unequivocal (evidence beyond reasonable doubt), credible (contains illustrations that may be challenged) or unsupported (when findings were not supported) [15, 17]. Second, findings of all included studies were extracted and categorised to create a set of categories representing meaningful similarities. Third, similar categories were further synthesised to obtain a comprehensive set of synthesised findings, which were used to develop evidence-based recommendations for practice. Two reviewers independently extracted findings, compared the generated categories and discussed discrepancies until reaching agreement.

### Assessing certainty in the finding

The final findings were graded according to the ConQual approach for establishing confidence in the output of research synthesis and presented in a summary of findings [17]. The ConQual process was used to analyse the level of confidence or trust that exists in the value and level of evidence of each synthesised finding as shown in Supplementary material 1.

## Results

Review results were reported in accordance with the ‘Enhancing Transparency in Reporting the Synthesis of Qualitative Research’ statement, which consists of 21 items and is appropriate for qualitative evidence synthesis (Supplementary file, Appendix 1).

### Study inclusion

**Fig. 1:**
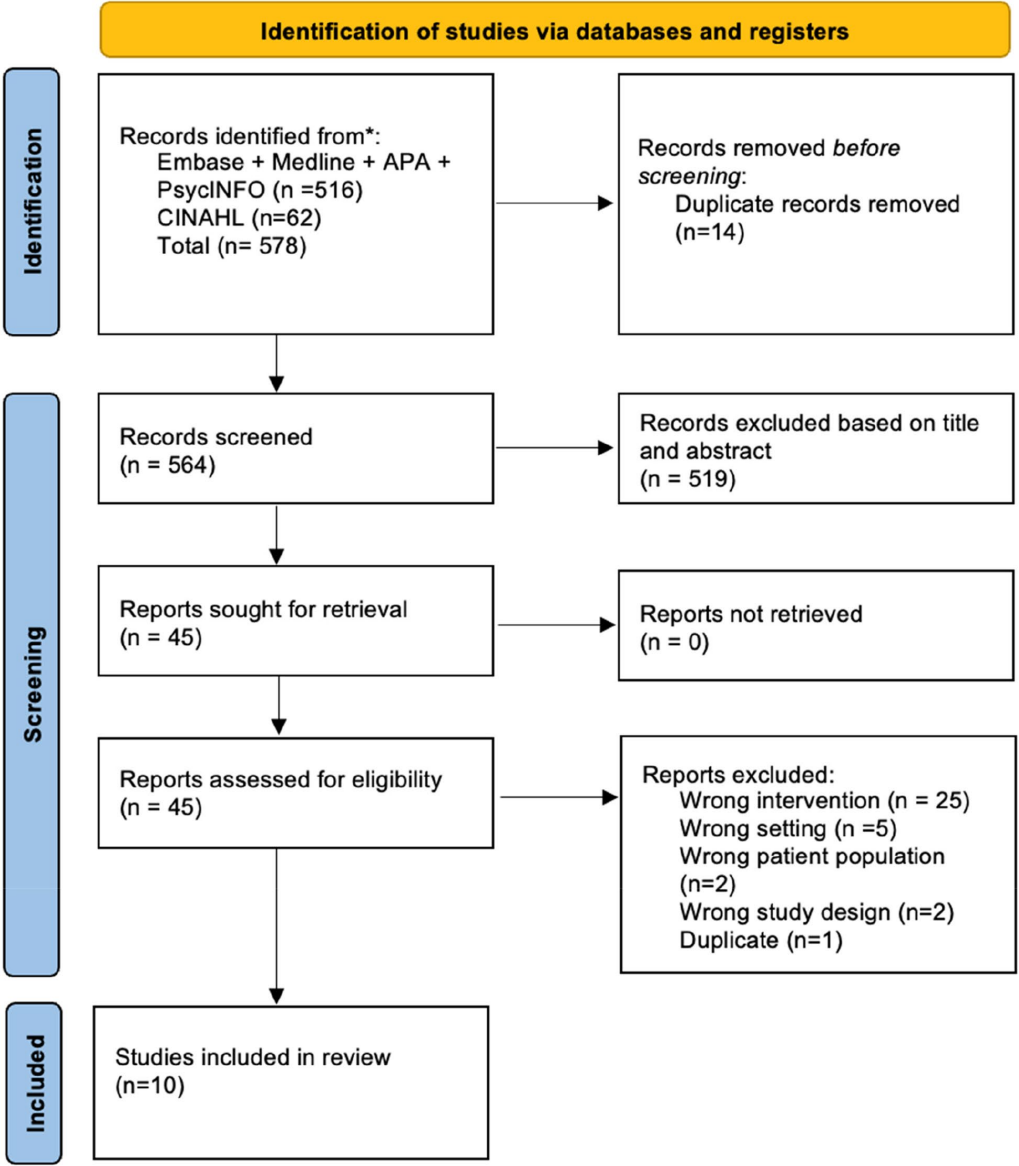
PRISMA chart flow diagram for inclusion of studies

The search strategy yielded 578 studies from MEDLINE, Embase, CINAHL and PsycINFO. A total of 14 articles were removed as they were duplicates which left 564 studies that were imported to JBI SUMARI. These studies were screened for eligibility by titles and abstracts, and 519 studies were excluded as they were not relevant to the topic. The remaining 45 studies were subject to further detailed assessment by examining the full text; 35 studies were excluded for reasons such as wrong phenomenon of interest, population, setting and/or study design. Overall, 10 studies were included in the review as shown in the Preferred Reporting Items for Systematic Review and Meta-Analysis (PRISMA) flow chart in Fig. 1.

### Methodological quality

From the assessment method of methodological quality, criteria 1, 3, 4 and 5 are all associated with the philosophical perspective, and congruity between the research methodology and methods used and the representation of analysis of the results were present in 8 of the 10 included studies except for O’Keefe et al. (2014) and Radcliff et al. (2009) as shown in Table 1 [11, 13]. Congruity between the research methodology and the research question in criteria 2 was present in all studies but two [11, 13]. In criteria 6, a statement locating the researcher culturally and theoretically was not included in three studies [10, 11, 18]. Criteria 7 examined the influence of the researcher on the research was addressed in all studies but one [11]. Additionally, the assessment of criteria 8 which relates to the representation of the participants voices was addressed in all the studies. Criteria 9 addressed ethics approval which were met in all the included studies. Finally, criteria 10 was associated with congruity between the study conclusion and the analysis and interpretation of the results which was present in all studies.

**Table 1:** Critical appraisal results, *qualitative research*

**Citation**	**Q1**	**Q2**	**Q3**	**Q4**	**Q5**	**Q6**	**Q7**	**Q8**	**Q9**	**Q10**
Astin F., Closs S. J., McLenachan J., Hunter S., Priestley C. (2009) [10]	Y	Y	Y	Y	Y	U	Y	Y	Y	Y
O’Keefe-McCarthy S., McGillion M., Nelson S., Clarke S. P., Jones J., Rizza S. et al. (2014) [11]	U	U	U	U	U	N	U	Y	Y	Y
Page M., Jackman K., Snowden P. (2008) [12]	Y	Y	Y	Y	Y	Y	Y	Y	Y	Y
Radcliffe E. L., Harding G., Rothman M. T., Feder G. S. (2009) [13]	U	U	U	U	U	Y	Y	Y	Y	Y
Sampson F., O’Cathain A., Goodacre S. (2009) [14]	Y	Y	Y	Y	Y	Y	Y	Y	Y	Y
Schroder S. L., Fink A., Richter M. (2018) [19]	Y	Y	Y	Y	Y	Y	Y	Y	Y	Y
Wilson T., Miller J., Teare S., Penman C., Pearson W., Marlett N. J. et al. (2017) [20]	Y	Y	Y	Y	Y	Y	Y	Y	Y	Y
Nakano A., Mainz J., Lomborg K. (2008) [21]	Y	Y	Y	Y	Y	Y	Y	Y	Y	Y
Andersson E. K., Skär L., Hjelm M. (2020) [22]	Y	Y	Y	Y	Y	Y	Y	Y	Y	Y
Bardsgjerde E. K., Kvangarsnes M., Landstad B., Nylenna M., Hole T. (2019) [18]	Y	Y	Y	Y	Y	N	U	Y	Y	Y
%	80.0	80.0	80.0	80.0	80.0	70.0	80.0	100.0	100.0	100.0

### Characteristics of included studies

The studies included in the review were published between 2008 and 2020, and all of them were from developed countries including the UK, Canada, Denmark, Sweden, Norway, Germany, and Australia. The participants included in the studies were patients who experienced an ACS and a hospital admission as a result of their illness. Patients age ranged between 35 and 80 years old. The sample size of the participants in the individual studies ranged from 4 to 41. A range of qualitative methodologies were used including semi-structured interviews and focus groups. The main phenomenon of interest across the studies was patient’s experience with their treatment following an ACS and management of their symptoms by health-care professionals. Some studies were specific to a particular procedure or aspect of the management of ACS such as angioplasty [13], pain management [11] and the remainder of the studies focused on the patients’ overall care experience. The setting of all studies were all metropolitan hospitals with one study set in a rural emergency department setting [11]. The characteristics of all included studies can be found in Table 2.

**Table 2:** Characteristics of included studies - interpretive and critical research form

**Study**	**Methods for data collection and analysis**	**Country**	**Phenomena of interest**	**Setting/context/culture**	**Participant characteristics and sample size**	**Description of main results**
Astin F., Closs S. J., McLenachan J., Hunter S., Priestley C. (2009) [10]	Semi-structured interviews and illness perception questionnaire	UK	Patient’s experience of primary angioplasty	Hospital	*n* = 29, age range 36-83, all participants admitted for the first time PCI and had no history of cardiac illness	Overall, participants were positive regarding their management; however, they experienced emotional shock which was further exacerbated by the speed by which events unfolded. There was an overall misalignment between their expectations of their treatment and the reality of what they were experiencing
O’Keefe-McCarthy S., McGillion M., Nelson S., Clarke S. P., Jones J., Rizza S. et al. (2014) [11]	Qualitative focus group and thematic analysis	Canada	Perspectives of ACS patients and emergency staff nurses on the rural experience of cardiac pain and anxiety as well as priorities and barriers to optimal assessment and management of ACS pain	Rural ED setting	Patients who had a recent ED admission in a rural hospital, *n* = 4	Patients described a significant wait time in the emergency room with regard to their initial assessment and management
Sampson F., O’Cathain A., Goodacre S. (2009) [14]	Semistructured interviews. framework analysis	UK	Views of patients undergoing primary angioplasty	Primary angioplasty patients	Patients with a STEMI undergoing	Patients were very positive about their experience with primary angioplasty. The speed and efficiency of the process and quick recovery time were impressive. The patients spoke about the procedure with a high level of confidence
Radcliffe E. L., Harding G., Rothman M. T., Feder G. S. (2009) [13]	Interviews and framework method of analysis	UK	Patients’ experience and perception of primary angioplasty as treatment for their heart attack	Tertiary hospital-cardiac unit	Patients admitted to a tertiary cardiology unit undergoing primary angioplasty (35-74 years) *n* = 15	Patients were impressed by noninvasive treatment they received in comparison to expectations of open-heart surgery. They were generally not engaged in decisions about the treatment in the acute setting. This passivity persisted until discharge
Nakano A., Mainz J., Lomborg K. (2008) [21]	Interviews and descriptive analysis	Denmark	What preoccupied patients admitted to cardiac care units with ACS and their perceptions on the care provided to them	Cardiac unit	Patients admitted to a cardiac unit with a diagnosis of ACS, *n* = 30	The patients felt they were in good hands. It was important to patients that they felt they were in good hands. The patients felt that staff were ready and waiting to assist them during the admission. The patients felt that the pain management was not always adequate. There was a large range of information provided to patients during the assessment ranging from chaotic to detailed and useful information. The patients were also not quite sure they could always remember the information they had been given. Patients also felt that the nursing staff provided compassionate and personalised care
Andersson E. K., Skär L. Hjelm M. (2020) [22]	Individual interviews- inductive analysis	Sweden	Cardiac care experiences of post-myocardial infarction	Hospital	Younger patients < 55 years old who had a myocardial infarction, *n* = 17	Patients were deemed to be in need tailored information with regard to their illness for their specific circumstances. Patients were also in need of recognition. They described a desire to discuss their personal illness experience and what happened to them with nurses to engage in personalised conversations with them regarding their situation. Patients needed a post-discharge rehabilitation plan. Many of the patients were not able to attend the patient education programme due to time restrictions. Thus, it was clear that an individualised post-discharge rehabilitation plan was needed
Bardsgjerde E. K., Kvangarsnes M., Landstad B., Nylenna M., Hole T. (2019) [18]	Interviews and narrative analysis	Norway	Patient experience of their participation in different phases of the myocardial infarction pathway	Hospitals without PCI facilities	Patients diagnosed with AMI, *n* = 10	The patients described a lack of verbal communication in the acute phase regarding their diagnosis and management plan—the patients revealed they trusted the healthcare professionals and the treatment they initially received. They described feeling in ‘safe hands’, and that the medical professionals were calm and knowledgeable in what to do in each situation. During the PCI procedure, patients reported that they received tailored information during or immediately after the PCI procedure. Patients described a lack of participation and coordination of care at discharge. There was a varied level of satisfaction and information provided with regard to lifestyle changes, further medical treatment and rehabilitation. Patients felt that there was shared decision-making in the rehabilitation phase of care
Wilson T., Miller J., Teare S., Penman C., Pearson W., Marlett N. J. et al. (2017) [20]	Focus groups and semi-structured interviews, grounded theory and descriptive analysis of text	Canada	Patient perspectives in engagement in decision-making in early management	Hospital	Patients with non-ST elevation acute coronary syndrome, *N* = 20	Participants found themselves lost in the process and especially when making decision regarding their management
Schroder S. L., Fink A., Richter M. (2018) [19]	Semi-structured interviews - comparative analysis	Germany	Socioeconomic differences in patient’s experiences with treatment of coronary heart diseases	Hospital	Elderly patients who suffered from coronary heart diseases aged (59-80) years old, *N* = 41	Three main themes were important to both groups, and they include amount of information provided, illness perception and level of decision-making in healthcare
Page M., Jackman K., Snowden P. (2008) [12]	Informal interviews, grounded theory, thematic analysis	Australia	The experiences of patients undergoing percutaneous transluminal coronary angioplasty	Hospital	Patients recruited from the coronary care unit, *N* = 11	Five key themes resulted from the interview analysis: these included misconceptions regarding causes of the disease; the overall procedure experience was not stressful; additional procedures could double concerns, pain and anxiety as a result of manual digital pressure and lack of post-discharge advice and support

### Review findings

**Fig. 2:**
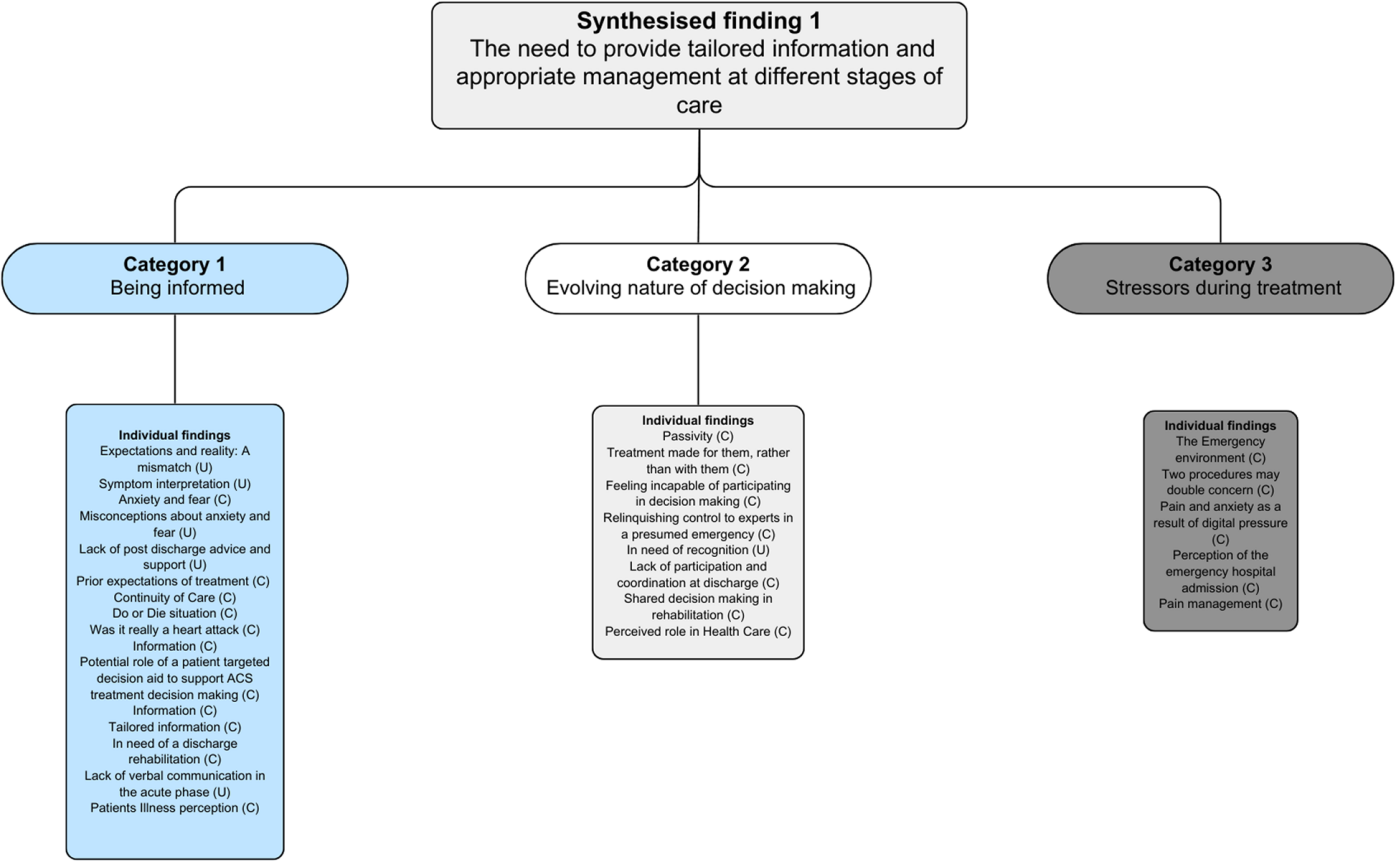
Synthesised findings 1

**Fig. 3:**
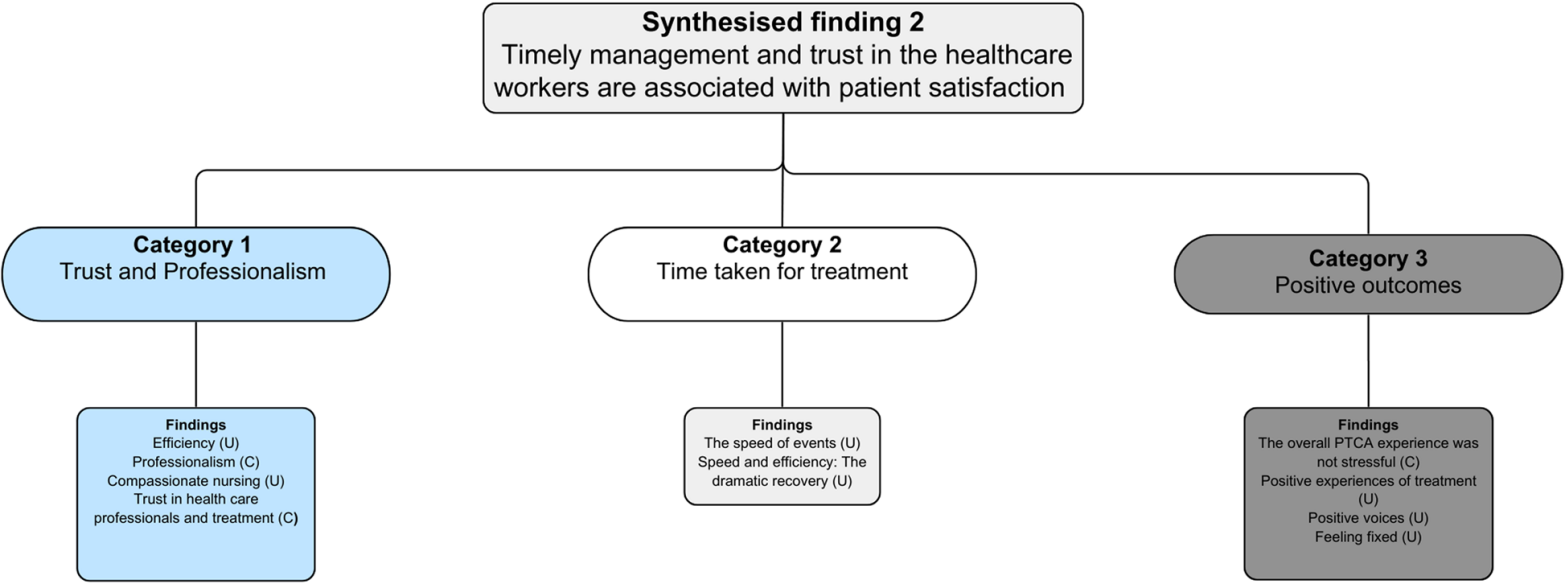
Synthesised findings 2

From the 10 included studies, 39 findings were extracted. Of these 39 findings, 26 were considered credible evidence, with the remaining 13 assigned as unequivocal evidence. All findings were further assigned to six categories based on their similarity. The categories were trust and professionalism, time taken for treatment, positive outcomes, being informed, evolving nature of decision-making and stressors during treatment. These six categories were then further combined through meta-aggregation into two overarching synthesised findings. The extracted findings for each study are shown in Appendix 1. The two synthesised findings were as follows: (1) the need to provide tailored information and appropriate management at different stages of care and (2) timely management and trust in health care workers are associated with patient satisfaction. A full overview of the findings linked to categories and synthesised findings can be seen in Figs. 2 and 3.

#### Synthesised findings 1: The need to provide tailored information and appropriate management at different stages of care

This synthesised finding was generated from the aggregation of three categories, underpinned by 29 extracted findings. The three categories were ‘being informed’, ‘evolving nature of decision making’ and ‘stressors during treatment’. Overall, patients felt stressed during their admission and were overwhelmed with the amount of information being related to them during their stay in hospital. They also appreciated receiving adequate information during their treatment, provided it is appropriate for each stage of their management. The first category ‘being informed’ is derived from 16 findings [9–13, 18–23]. These findings related to the provision of tailored information. Some of the findings were related to lack of verbal communication in the acute phase, and others were related to fear, anxiety and their expectations of the treatment and the need to know about the plan of their rehabilitation and the information needed to ensure their continuity of care. The second category ‘evolving nature of decision making’ was derived from eight findings [13, 18–20, 22]. These findings were related to some patients feeling that the treatment was being done for them rather than with them. Some patients felt too sick and were incapable of making decisions. The third category ‘stressors during treatment’ was derived from five findings [12, 13, 23]. These findings were related to the stressful environment of the emergency department, the pain and anxiety during manual examination and the procedures patients were undergoing.

#### Synthesised findings 2: Timely management and trust in health care workers are associated with patient satisfaction

This synthesised finding was generated from the aggregation of three categories, underpinned by 10 extracted findings. These three categories were ‘trust and professionalism’, ‘time taken for treatment’ and ‘positive outcomes’. Overall, patients’ satisfaction was dependent on health care professionals showing compassion, care, time spent with patients, professionalism and the positive outcomes they are experiencing as a result of their management. The first category ‘trust and professionalism’ was derived from four categories related to efficiency, professionalism, compassion and trust [18, 21]. These findings capture the appreciation that patients have towards health care professionals when patients feel that they are cared for.

The second category ‘time taken for treatment’ is derived from two findings related to the speed of events and the efficiency of management which results in a dramatic recovery [10, 14]. This category captures how patients appreciate the time taken to be managed efficiently by the health care team.

The third category ‘positive outcomes’ is derived from four findings related to positive experiences, positive voices and patients feeling fixed [12–14, 23]. These findings capture the positive feelings associated with being well and getting better.

## Discussion

This systematic review detailed patients’ experience and perception with healthcare provision following an ACS. Overall, there were two main synthesised findings generated from the included studies. Firstly, that patients appreciate tailored information being provided during the different stages of care as well as being involved in their management and decisions regarding their care. This includes not only during their initial admission and treatment phase but also for longer term after discharge to ensure continuity of care [22, 24, 25]. The second synthesised finding related to the trust, care, professionalism and time spent with them by the health professionals responding to their needs. This finding highlights the enormous stress patients experience following their initial hospital admission as well as the changing nature of their condition over time. These two findings can be broadly categorised as factors related to the health care professional and the hospital culture [7]. The professional and personal characteristics of health care professionals included the trust, compassion, knowledge and professionalism that were valued by the patients. The hospital culture included factors such as the efficiency of the system, the information provided to patients and the shared decision-making process employed by the team in managing the patients [26–28].

Understanding the challenges and needs faced by patients after an ACS, particularly during their hospital admission, is essential to determine the most appropriate patient-centred management plan for each individual patient [18]. Patient centred and individualised care can improve patient satisfaction and impact on overall patient experience which is important to both health care providers and service providers [29]. This in turn can contribute to improved care and reduce medical burden and unnecessary treatment [29]. The provision of hospital service quality is associated with patient satisfaction [30]. Swain and Kar (2018) identified six main areas through which patients perceive quality of service in hospitals. These included staff attitude, personalised attention, information availability, staff competency, trustworthiness and waiting time as essential to their overall satisfaction with the care they receive [31]. These areas are important for patients when considering that they are receiving good quality care from a health service provider.

The findings of this review are consistent with other studies investigating patients’ experience and satisfaction with their care. In one study in Singapore, authors undertook an integrative review including both quantitative and qualitative studies and found that patients’ experience and satisfaction were dependent on nurses caring behaviour and the amount of information provided to patients by them for their management [32]. The authors also found that patients appreciated prompt responses to their needs and appreciated technical information given to them [32].

Another quantitative systematic review found several factors to be contributing to positive patients experience with their care [20]. These included specific health care provider factors such as their professionalism, competency and interpersonal care. The authors also found that the physical environment the patients are placed in was another contributing factor to their overall experience. Efficacy and the outcome of the care were also identified as strong predictors of patients’ positive experience [33]. These findings correspond with the findings reported in this review as patients experiencing positive outcomes of their care showed high satisfaction. Targeting educational interventions to support health care professionals’ behaviours towards patient care and highlighting what issues are important to patients are a major determinant of patient satisfaction [12]. As for health service providers, initiating policies and guidelines aiming at involving patients in their decision-making and ensuring the provision of timely management to patients is also another important determinant of patient satisfaction with their care [10, 23].

This review highlights several important implications for future clinical practice. Clinicans should be aware and carefully consider the delivery of appropiate information to patients during their care and patient involvement in their own management. Additionally, clinicans should aim to create an enviroment of trust and professionalism, where they aim to respond to patients’ needs and concerns in a timely and efficient manner.

A limitation of this review is that the findings may not be generalisable to all countries as all the included studies were undertaken in western countries. Other countries may experience different challenges not mentioned in the included studies. Further research on this topic is needed in non-Western countries.

## Conclusions

Patients with acute coronary syndrome experience many challenges during their health care journey. This review has found that patients need tailored information given that is appropriate for their different stages of management. In addition, patients appreciate and want to be involved in their care and be part of the decision-making process. Health care professionals should make time to identify and address patient needs which will result in increased patient satisfaction and a positive patient experience.

### Supplementary Information

Supplementary Material 1. Search strategy. Database. CINAHL search strategy. JBI Critical Appraisal Checklist for Qualitative Research. Table 1 Summary of the Enhancing Transparency in Reporting the Synthesis of Qualitative Research statement. Table 2. List of Study Findings with Illustrations U-unequivocal, C-credible.

## References

[1] Mohseni J, Kazemi T, Maleki MH, Beydokhti H (2017). A systematic review on the prevalence of acute myocardial infarction in Iran. Heart Views.

[2] WHO. WHO reveals leading causes of death and disability worldwide: 2000-2019 2020. Available from: https://www.who.int/news/item/09-12-2020-who-reveals-leading-causes-of-death-and-disability-worldwide-2000-2019.

[3] Dafaalla M, Rashid M, Weston C, D'Ascenzo F, De Ferrari GM, Hussain ST (2021). Effect of the timing of admission of out of hospital cardiac arrest complicating acute myocardial infarction on management and outcome. Am J Cardiol.

[4] Kumah E, Patient experience and satisfaction with a healthcare system: connecting the dots. Int J Healthc Manag. 2019;12(3):173-79.

[5] Omar S, Morgan GL, Panchal HB, Thourani V, Rihal CS, Patel R (2018). Management of post-myocardial infarction ventricular septal defects: a critical assessment. J Interv Cardiol.

[6] Anhang Price R, Elliott MN, Zaslavsky AM, Hays RD, Lehrman WG, Rybowski L (2014). Examining the role of patient experience surveys in measuring health care quality. Med Care Res Rev.

[7] Mazurenko O, Collum T, Ferdinand A, Menachemi N (2017). Predictors of hospital patient satisfaction as measured by HCAHPS: a systematic review. J Healthc Manag.

[8] Doyle C, Lennox L, Bell D (2013). A systematic review of evidence on the links between patient experience and clinical safety and effectiveness. BMJ Open.

[9] Larson E, Sharma J, Bohren MA, Tunçalp Ö (2019). When the patient is the expert: measuring patient experience and satisfaction with care. Bull World Health Organ.

[10] Astin F, Closs SJ, McLenachan J, Hunter S, Priestley C (2009). Primary angioplasty for heart attack: mismatch between expectations and reality?. J Adv Nurs (Wiley-Blackwell)..

[11] O’Keefe-McCarthy S, McGillion M, Nelson S, Clarke SP, Jones J, Rizza S (2014). Acute coronary syndrome pain and anxiety in a rural emergency department: patient and nurse perspectives. Can J Nurs Res..

[12] Page M, Jackman K, Snowden P (2008). The experiences of patients undergoing percutaneous transluminal coronary angioplasty: a qualitative exploration. CONNECT: World Crit Care Nurs.

[13] Radcliffe EL, Harding G, Rothman MT, Feder GS (2009). ‘It got right to the spot’ The patient experience of primary angioplasty: a qualitative study. Eur J Cardiovasc Nurs.

[14] Sampson F, O'Cathain A, Goodacre S (2009). Feeling fixed and its contribution to patient satisfaction with primary angioplasty: a qualitative study. Eur J Cardiovasc Nurs.

[15] Lockwood C, Munn Z, Porritt K (2015). Qualitative research synthesis: methodological guidance for systematic reviewers utilizing meta-aggregation. JBI Evid Implement.

[16] Munn Z, Aromataris E, Tufanaru C, Stern C, Porritt K, Farrow J (2019). The development of software to support multiple systematic review types: the Joanna Briggs Institute System for the Unified Management, Assessment and Review of Information (JBI SUMARI). JBI Evid Implement.

[17] Munn Z, Porritt K, Lockwood C, Aromataris E, Pearson A (2014). Establishing confidence in the output of qualitative research synthesis: the ConQual approach. BMC Med Res Methodol.

[18] Bardsgjerde EK, Kvangarsnes M, Landstad B, Nylenna M, Hole T (2019). Patients’ narratives of their patient participation in the myocardial infarction pathway. J Adv Nurs.

[19] Schroder SL, Fink A, Richter M (2018). Socioeconomic differences in experiences with treatment of coronary heart disease: a qualitative study from the perspective of elderly patients. BMJ Open.

[20] Wilson T, Miller J, Teare S, Penman C, Pearson W, Marlett NJ (2017). Patient perspectives on engagement in decision-making in early management of non-ST elevation acute coronary syndrome: a qualitative study. BMC Med Inform Decis Mak.

[21] Nakano A, Mainz J, Lomborg K (2008). Patient perception and assessment of admission to acute cardiac care unit. Eur J Cardiovasc Nurs.

[22] Andersson EK, Skär L, Hjelm M (2020). Care experiences of younger people and next of kin following myocardial infarction. Br J Cardiac Nurs.

[23] Sampson FC, O'Cathain A, Goodacre S, Astin ABBVCCCDDEFGGKOCOCRRST (2010). Is primary angioplasty an acceptable alternative to thrombolysis? Quantitative and qualitative study of patient and carer satisfaction. Health Expect.

[24] Crawshaw J, Bartoli-Abdou JK, Weinman J, McRobbie D, Stebbins M, Brock T (2021). The transition from hospital to home following acute coronary syndrome: an exploratory qualitative study of patient perceptions and early experiences in two countries. Int J Pharm Pract.

[25] Lowe E, Banner D, Estefan A, King-Shier K (2022). Being uncertain: rural-living cardiac patients’ experience of seeking health care. Qual Health Res.

[26] Vingerhoets C, Hay-Smith J, Graham F. Getting to know our patients and what matters: exploring the elicitation of patient values, preferences, and circumstances in neurological rehabilitation. Disabil Rehabil,. 2023;45(9):1444-52. 10.1080/09638288.2022.2063416 35476588

[27] Grundnig JS, Steiner-Hofbauer V, Katz H, Holzinger A (2022). ‘Good’and ‘bad’doctors-a qualitative study of the Austrian public on the elements of professional medical identity. Med Educ Online.

[28] Simpson AV, Farr-Wharton B, Reddy P (2020). Cultivating organizational compassion in healthcare. J Manag Organ.

[29] Manzoor F, Wei L, Hussain A, Asif M, Shah SIA (2019). Patient satisfaction with health care services; an application of physician’s behavior as a moderator. Int J Environ Res Public Health.

[30] Ganasegeran K, Perianayagam W, Abdul Manaf R, Ali Jadoo SA, Al-Dubai SAR (2015). Patient satisfaction in Malaysia’s busiest outpatient medical care. ScientificWorldJournal..

[31] Swain S, Kar NC. Hospital service quality as antecedent of patient satisfaction–a conceptual framework. Int J Pharm Healthc Market. 2018;12.

[32] GOH ML, Vehviläinen-Julkunen K. Hospitalised patients’ satisfaction with their nursing care: an integrative review. Singapore Nurs J. 2016;43(2):11–27.

[33] Batbaatar E, Dorjdagva J, Luvsannyam A, Savino MM, Amenta P (2017). Determinants of patient satisfaction: a systematic review. Perspect Public Health.

