# Supplementary material for: Experiences and challenges of acute coronary syndrome patients in care provision: a qualitative systematic review
Source: Syst Rev. 2024 Jul 17;13:184. doi: 10.1186/s13643-024-02578-1 (PMC11253477; doi:10.1186/s13643-024-02578-1)
Supplement: Supplementary file 1 — Supplementary Material 1. Search strategy. Database. CINAHL search strategy. JBI Critical Appraisal Checklist for Qualitative Research. Table 1 Summary of the Enhancing Transparency in Reporting the Synthesis of Qualitative Research statement. Table 2. List of Study Findings with Illustrations U-unequivocal, C-credible. [file 13643_2024_2578_MOESM1_ESM.docx]

**Appendix 1**

**Search strategy**

**Database:**

Embase Classic+Embase <1947 to 2022 July 13>

Ovid MEDLINE(R) <1946 to July 13, 2022>

APA PsycInfo <1806 to July Week 2 2022>

| **#** | **Query** | **Results from 14 Jul 2022** |
| --- | --- | --- |
| 1 | (myocardial infarction or ST elevation myocardial infarction or non-ST elevation myocardial infarction or acute coronary syndrome).mp. [mp=ti, ab, hw, tn, ot, dm, mf, dv, kf, fx, dq, nm, ox, px, rx, ui, sy, tc, id, tm] | 651,921 |
| 2 | (experience or satisfaction or provision or quality).mp. [mp=ti, ab, hw, tn, ot, dm, mf, dv, kf, fx, dq, nm, ox, px, rx, ui, sy, tc, id, tm] | 6,436,758 |
| 3 | (health care or medical care or acute care or primary care or hospital care or health service$ care).mp. [mp=ti, ab, hw, tn, ot, dm, mf, dv, kf, fx, dq, nm, ox, px, rx, ui, sy, tc, id, tm] | 3,238,763 |
| 4 | (focus group or qualitative or fieldwork or face to face or interview).mp. [mp=ti, ab, hw, tn, ot, dm, mf, dv, kf, fx, dq, nm, ox, px, rx, ui, sy, tc, id, tm] | 1,633,549 |
| 5 | 1 and 2 and 3 and 4 | 543 |
| 6 | limit 5 to english language | 529 |
| 7 | limit 6 to humans [Limit not valid in APA PsycInfo; records were retained] | 516 |

(myocardial infarction or ST elevation myocardial infarction or non-ST elevation myocardial infarction or acute coronary syndrome).mp. [mp=ti, ab, hw, tn, ot, dm, mf, dv, kf, fx, dq, nm, ox, px, rx, ui, sy, tc, id, tm]

(experience or satisfaction or provision or quality).mp. [mp=ti, ab, hw, tn, ot, dm, mf, dv, kf, fx, dq, nm, ox, px, rx, ui, sy, tc, id, tm]

(health care or medical care or acute care or primary care or hospital care or health service$ care).mp. [mp=ti, ab, hw, tn, ot, dm, mf, dv, kf, fx, dq, nm, ox, px, rx, ui, sy, tc, id, tm]

(focus group or qualitative or fieldwork or face to face or interview).mp. [mp=ti, ab, hw, tn, ot, dm, mf, dv, kf, fx, dq, nm, ox, px, rx, ui, sy, tc, id, tm]

1 and 2 and 3 and 4

limit 5 to english language

limit 6 to humans

**CINAHL search strategy**


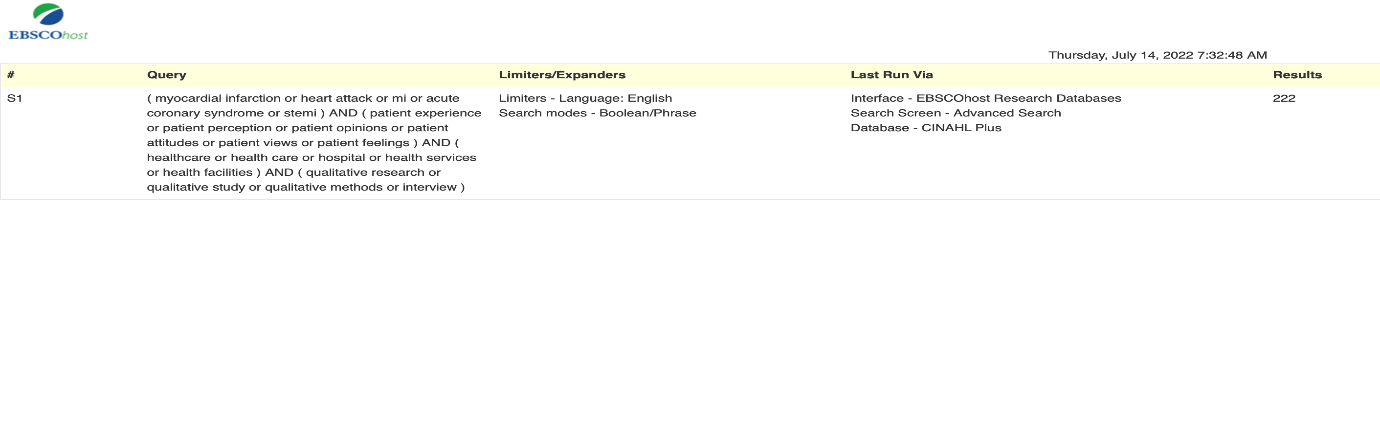


**JBI Critical Appraisal Checklist for Qualitative Research**

Reviewer ______________________________________ Date_______________________________

Author_______________________________________ Year_________ Record Number_________

|  | Yes | No | Unclear | Not applicable |
| --- | --- | --- | --- | --- |
| 1. Is there congruity between the stated philosophical perspective and the research methodology? | □ | □ | □ | □ |
| 1. Is there congruity between the research methodology and the research question or objectives? | □ | □ | □ | □ |
| 1. Is there congruity between the research methodology and the methods used to collect data? | □ | □ | □ | □ |
| 1. Is there congruity between the research methodology and the representation and analysis of data? | □ | □ | □ | □ |
| 1. Is there congruity between the research methodology and the interpretation of results? | □ | □ | □ | □ |
| 1. Is there a statement locating the researcher culturally or theoretically? | □ | □ | □ | □ |
| 1. Is the influence of the researcher on the research, and vice- versa, addressed? | □ | □ | □ | □ |
| 1. Are participants, and their voices, adequately represented? | □ | □ | □ | □ |
| 1. Is the research ethical according to current criteria or, for recent studies, and is there evidence of ethical approval by an appropriate body? | □ | □ | □ | □ |
| 1. Do the conclusions drawn in the research report flow from the analysis, or interpretation, of the data? | □ | □ | □ | □ |

Overall appraisal: Include □ Exclude □ Seek further info □

Comments (Including reason for exclusion)

| **Table 1** Summary of the Enhancing Transparency in Reporting the Synthesis of Qualitative Research statement | |
| --- | --- |
| **No** | **Item** |
| 1 | Aim |
| 2 | Synthesis methodology |
| 3 | Approach to searching |
| 4 | Inclusion criteria |
| 5 | Data sources |
| 6 | Electronic search strategy |
| 7 | Study screening methods |
| 8 | Study characteristics |
| 9 | Study selection results |
| 10 | Rationale for appraisal |
| 11 | Appraisal items |
| 12 | Appraisal process |
| 13 | Appraisal results |
| 14 | Data extraction |
| 15 | Software |
| 16 | No of reviewers |
| 17 | Coding |
| 18 | Study comparison |
| 19 | Derivation of themes |
| 20 | Quotations |
| 21 | Synthesis output |

**Table 2. List of Study Findings with Illustrations**

**U-unequivocal, C-credible**

| Study: Astin 2009 | |
| --- | --- |
| Finding | The speed of events (U) |
| Illustration | within two hours, I was lying in a bed recovering after what they 'd done, it was amazing, a bit of a shock |
| Finding | Expectations and reality: a mismatch (U) |
| Illustration | ‘As I say, I was just expecting (laughing), I mean, I don’t want to go down to the hospital just to scar me up a bit, like (laughing). But, er, no, I expected a bit more, bit more pain more pain and a lot more (short pause) bit more blood...it’s a lot like nothing’s happened. |
| Study: O'Keefe-McCarthy 2014 | |
| Finding | symptom interpretation (U) |
| Illustration | I’ve had pain so many times, this chest pain . . . but the pain, whether it’s been my heart, or maybe my chronic back pain or what- ever . . . you’re wondering which it is. |
| Finding | Anxiety and fear (C) |
| Illustration | You’re worried when you’re first there [in the ED] and you’re feeling all the pain and — okay . . . what’s going to happen now? |
| Finding | The Emergency Environment (C) |
| Illustration | spent 3 days waiting. You wait for your angiogram because there’s a time delay. |
| Study: Page 2008 | |
| Finding | Misconceptions about causes of their condition (U) |
| Illustration | No-one said why it happened. I still can’t understand why I got it. It was the last thing I expected |
| Finding | The overall PTCA experience was not stressful (C) |
| Illustration | It’s a commonplace thing... to me nowadays, having a heart attack is not much different to having a common cold (Male, 58) |
| Finding | Two procedures may double concerns (C) |
| Illustration | One of my wife’s friends who had the same thing said to me don’t have two procedures, make sure they only do one procedure. Her husband had it and they sent him home after the angiogram to come back for the stents. He dropped down dead the next morning |
| Finding | Pain and anxiety as a result of manual digital pressure (C) |
| Illustration | When they had to put that ball thing in and puff it up to stop the bleeding. That was the biggest discomfort I had. That hurt, that was quite sore, but that’s alright. |
| Finding | Lack of post discharge advice and support (U) |
| Illustration | Just behave myself mate. That’s what I was told. Take it easy. |
| Study: Radcliffe 2009 | |
| Finding | prior expectations of treatment (C) |
| Illustration | “I didn't expect anything like I had, what is it, angioplasty? I've never heard of it. I thought it was open heart surgery. |
| Finding | Positive experiences of treatment (U) |
| Illustration | I've had an A1 service from the NHS full stop |
| Finding | Passivity (C) |
| Illustration | It's rather like if your car breaks down, and the AA man comes round, he knows best, you're not going to tell him “well I can tell you what the matter is”, and it's the same with hospitals, once you go through the door, I'm quite happy, more than happy. |
| Finding | Continuing care (C) |
| Illustration | I think if they could maybe give one visit, just to see if the medication is okay and see how you are feeling but not to send anybody and just left in the dark about what tablets, or what you're meant to do. It's just ridiculous really |
| Study: Sampson 2009 | |
| Finding | Positive voices (U) |
| Illustration | It is brilliant, really brilliant. He couldn't have got any better treatment if he, if he was the queen I don't think. |
| Finding | ‘Do or die’ situation (C) |
| Illustration | Hesaid‘I'mDrsoandso,IwilltreatyoubutIwantyou to go to [hospital 1]. But you must give your consent to what they do’. Well I had no idea what he was talking about so I said yeah alright. |
| Finding | Speed and efficiency: the dramatic recovery (U) |
| Illustration | The thing that I, that I was very impressed with and perhaps amazed me, having read certain negative things in news- papers etc, which one shouldn't read, was the, the speed and efficiency at which this just came together |
| Finding | Feeling fixed (U) |
| Illustration | I mean, within three hours of the heart attack, I was back in a ward, back in a bed, basically fixed. |
| Finding | Was it really a heart attack? (C) |
| Illustration | Whether I had a heart attack or whether it was a near thing I'm not even sure now. I've been told I had a heart attack but it is not what I thought I would have had |
| Study: Schroder 2018 | |
| Finding | Information (C) |
| Illustration | And as well, someone who is cognitively not that fit anymore, one does not understand at all what you are told. And that is sometimes not so nice. Well, but probably that’s just our system, that is/which is not working, I think. ( |
| Finding | Patients' Illness perception (C) |
| Illustration | Well, in the hope that everything stays the same. Let’s say that you are well. Well, getting around with it quite well, with no complications arising. As I said, that one may reach a slightly older age, not just until retirement, but perhaps even a bit longer. |
| Finding | Perceived role in healthcare (C) |
| Illustration | Today, quite a few medical exams have been conducted with me. Now you have to wait, but I guess it won’t change anything about this final decision. The demand placed on me to decide this. |
| Study: Wilson 2017 | |
| Finding | Perception of the emergency hospital admission (C) |
| Illustration | “This was the most significant emotional event in my life” …“a wake-up call”. “It hit me square in the head”…“not being invincible was the biggest shock” |
| Finding | Treatments made for them, rather than with them (C) |
| Illustration | They’ve looked at the options and have decided what is best for you and then they tell you that and you decide if you will do it or not |
| Finding | Feeling incapable of participating in decision-making (C) |
| Illustration | They took the decision away from me, and I’m glad as that reduced my stress |
| Finding | Relinquishing control to experts in a presumed emergency (C) |
| Illustration | I expected the surgeon knew what he was doing and did exactly what he said he would do…No question there |
| Finding | Potential role of a patient-targeted decision aid to support ACS treatment decision-making (C) |
| Illustration | Information isn’t volunteered….you have to know what to ask |
| Study: Nakano 2008 | |
| Finding | efficiency (U) |
| Illustration | It was chaos and confusion. There were so many faces. I thought to myself, what a lot of people waiting for me. It was fantastic. They put the drops up really quickly, here and here. I don't know whether I had actually expected that you would be waiting for me. Everything was ready for me in the emergency room. I can't remember very much, but all of a sudden a lot happened very quickly. It was very efficient. |
| Finding | Professionalism (C) |
| Illustration | I can't quite remember what the nurse who admitted me said nor did, I just can't remember. They strapped me up to all sorts of things, on my legs and arms, on my chest and stomach. I had plasters all over me. When they explained what was going on… I just nodded and that was that. But it wasn't anything I thought about. They did what they did. Very well-planned and skilled work … I felt that they all knew what to do. |
| Finding | Pain management (C) |
| Illustration | They started on me as soon as I arrived. I was very happy with it. I was writhing around a bit. It still hurt up here. They gave me a jab, but it didn't really help. They gave me more jabs and put up a drop and everything. It was as though everything was swimming before my eyes…it started hurting terribly again. So I don't really remember all the details. |
| Finding | Compassionate nursing (U) |
| Illustration | The nurses were wonderful. It was great and I admire your colleagues very much. I knew very well that it was close to the end of their duty and they were still fantastic. I couldn't put a finger on anything. They were very caring — it was fantastic. They were so pleasant and helpful and concerned about me. It was as though I wasn't just a number. I didn't feel that. It was just great. |
| Finding | Information (C) |
| Illustration | No, I never found out who was responsible for me. I don't know whether there were a lot of people. I just lay still and stared at the ceiling. I had been told to lie absolutely still and keep quiet. I tried to. No, I don't remember. |
| Study: Andersson 2020 | |
| Finding | Tailored information (C) |
| Illustration | I asked the doctor why, why I got it (myocardial infarction). “Oh, it was just bad luck that it was you,” he said basically, because I don't smoke or take snuff or have any family history of it, so to say. So he really didn't have any explanation for why me. |
| Finding | In need of recognition (U) |
| Illustration | I think that the healthcare professionals should see more, to the extent that it is possible to individualise, so that they work more person-centred, according to my needs and how I do after this (myocardial infarction). What status am I in, when I come back from the illness? What do I need? |
| Finding | In need of a post-discharge rehabilitation plan (C) |
| Illustration | for the ones of us who are younger, there must be some individual opportunities, e.g. in the evening, because it's so important this Heart School and gymnastics and all of that, to also have some opportunities in the evenings for those of us who can't go during the day. We have such individual prerequisites and goals. Because it's really stressful having to take time off from work. I mean, it's not always appropriate to do that once a week and the gymnastics is two times a week, like the last one that is two times a week. |
| Study: Bardsgjerde 2019 | |
| Finding | Lack of verbal communication in the acute phase (U) |
| Illustration | They did not say anything. Except the chief physician saying; you will be transferred immediately |
| Finding | Trust in healthcare professionals and treatment (C) |
| Illustration | It seemed like it was something that they had done several times before and that it actually was not a severe intervention |
| Finding | Lack of participation and coordination at discharge (C) |
| Illustration | If you could receive simple diet advices at the hospital, for example, reduce the use of milk products and choose oils instead of butter, then you could start make changes already at discharge |
| Finding | Shared decision‐making in rehabilitation (C) |
| Illustration | I will never have another myocardial infarction. I know you cannot choose that, but I will do my very best to prevent another one |
